# SNP-Based KASP Markers Reveal Genetic Diversity and Population Structure Among African Sorghum Breeding Lines and Hybrids

**DOI:** 10.3390/plants15162488

**Published:** 2026-08-17

**Authors:** Caleb Mugove Souta, Zamalotshwa Goodness Thungo, Julia Sibiya, Pangirayi Tongooona, Meluleki Zikhali

**Affiliations:** 1 College of Agriculture, Engineering and Science (CAES), University of KwaZulu-Natal (UKZN), Private Bag X01, Scottsville, Pietermaritzburg 3209, South Africa; caleb.souta@seedcogroup.com (C.M.S.);; 2 Seed Co., Ltd., Rattray Arnold Research Station, Chisipite, Harare P.O. Box CH142, Zimbabwe

**Keywords:** genetic diversity, heterotic grouping, population structure, KASP, SNP, *Sorghum bicolor*

## Abstract

Sorghum is a strategic food-security and livestock-feed crop in Africa, and molecular characterization of its germplasm underpins genetic diversity assessment and hybrid parent selection. This study evaluated 106 sorghum accessions (23 A-lines, 28 B-lines, 32 R-lines, and 23 hybrids) from ICRISAT and Seed Co gene banks using 79 single nucleotide polymorphism (SNP)-based Kompetitive Allele-Specific PCR (KASP) markers. Genetic diversity, population structure, cluster analysis, and analysis of molecular variance (AMOVA) were assessed to support heterotic grouping. Gene diversity ranged from 0.23 in R-lines to 0.32 in hybrids, and polymorphic information content from 0.19 to 0.26, indicating moderate marker discriminatory power. Population structure analysis identified an optimum of K = 3: Cluster 1 comprised paired A- and B-lines forming a putative maintainer pool, Cluster 2 was dominated by R-lines forming a divergent restorer pool, and Cluster 3 formed a second seed-parent subgroup. AMOVA confirmed highly significant differentiation (ΦST = 0.55; *p* < 0.001), with molecular variance partitioned among the three clusters (44.0%), among individuals within clusters (45.2%), and among breeding-line types (10.8%). These findings provide a genomic framework for assigning sorghum accessions to putative heterotic groups and accelerating hybrid parent selection for climate-resilient sorghum production.

## 1. Introduction

Sorghum [*Sorghum bicolor* (L.) Moench, 2n = 2x = 20] is one of the primary grains in the world after wheat, rice, barley, and maize. It is staple to millions of the poorest and most food insecure people in the semi-arid and dry regions of Africa and Asia [1]. Other than food security, sorghum supports animal feed, fiber and biofuel production [2]. The crop’s natural adaptability to environmental stress, including heat and drought, makes it ideal for cultivation in semi-arid and arid regions [3,4]. These suggest the requirement for enhanced cultivation of sorghum to optimize global food and nutrition security. The main obstacles to an increase in sorghum production are the lack of improved varieties that suit erratic climate change, depleted arable land, and reduced water resources, which play a critical role in increasing global crop productivity [5]. As a result, there is an increasing need in cultivar design and development to enhance sorghum productivity under diverse production environments.

Sorghum possesses remarkable genetic variation accumulated over natural selection, domestication events, and adaptation to diverse production environments [6,7]. Genetic variation is pivotal in crop improvement, key to providing a wide selection base for breeding material that possess resilience and enhanced productivity [8]. Ethiopia is believed to be one of the centers of origin of sorghum; therefore, many races and their associated intermediates are grown throughout the nation’s agro-ecological zones and farming systems [9]. It is well known that the rich genetic variability seen in Ethiopian sorghum germplasm is a source of beneficial genes for improving sorghum [10]. Nevertheless, the full potential of sorghum’s genetic diversity remains insufficiently characterized and underutilized in modern crop improvement programs. Effective utilization of the crop’s genetic diversity requires accurate characterization of genetic relationships and population structure in cultivated sorghum varieties, which is fundamental to aid heterotic groups and breeding population development [11].

The three-line system, i.e., A-lines, maintainer B-lines, and restorer R-lines involved in the development of cytoplasmic male sterility, is central to the accumulation of yield gains in commercial sorghum production [5,12,13,14]. Therefore, the molecular characterization of A-, B-, and R-lines to understand genetic diversity among and within these lines enables breeders to develop a broader genetic base and identify novel maintainer and restorer lines. Molecular characterization in sorghum has been implemented through various types of molecular markers. Markers such as amplified fragment length polymorphism (AFLPs), restriction fragment length polymorphism (RFLPs), diversity arrays technology (DArTs), randomly amplified polymorphism DNA (RAPDs), and simple sequence repeats (SSRs) have been used [15]. The single-nucleotide polymorphisms (SNP) markers are currently at the peak due to high abundance and can accommodate the whole genome with high throughput and high resolution compared to others [16]. Using fluorescent labelled allele-specific primers for bi-allelic SNP discrimination, the Kompetitive Allele-specific PCR (KASP) markers offer high accuracy, efficiency and flexibility in genotyping [17]. The SNP-based KASP markers were validated in sorghum [18], confirming their precision for genetic diversity analysis at target loci.

Population structure analysis is fundamental for effective germplasm characterization in plant breeding, partitioning genotypes into unique sub-groups based on patterns of allelic sharing. This provides a reflection for shared ancestry, geographic origin, and accumulative effects of selection, genetic drift, and gene flow [19]. The application of population structure analysis has delineated genetically diverse sub-populations and assigned breeding materials into clusters based on membership coefficient values [11]. The delta K method enables the determination of an optimum number of genetic clusters, which identifies the most probable number of sub-populations [20]. In hybrid sorghum breeding, population structure analysis provides verification of the genetic integrity of A-, B-, and R-line gene pools, confirming the expected divergence between maintainer and restorer gene pools, and identifying misclassified accessions that may reduce the efficiency of the three-line hybrid system.

Cluster analysis groups accessions based on genetic distance, facilitating identification of genetically similar and divergent germplasm subsets. The use of approaches such as neighbor-joining dendrograms, the unweighted pair group method with arithmetic mean (UPGMA) clustering, and model-based STRUCTURE analysis have been widely applied in sorghum [21]. The genome-wide SNP-based clustering resolves sorghum accessions into biologically meaningful groups corresponding to racial classifications and geographic origins [22]. In hybrid breeding, cluster analysis supports the assignment of parental lines to distinct heterotic groups, enabling identification of divergent combinations most likely to generate superior hybrids [23]. The analysis of molecular variance (AMOVA) balances population structure and cluster, providing a ranked decomposition of total genetic variation into components. The components are attributable to differences among populations, among individuals within populations, and within individuals [24]. Applied to A-, B-, and R-line gene pools, AMOVA quantified genetic differentiation among functionally distinct breeding groups and guided the selection of complementary hybrid parent combinations [25]. The aim of this study was to evaluate the genetic diversity and population structure among selected sorghum A-, B-, and R-line accessions using SNP-based KASP markers, to generate genomic information supporting heterotic grouping for genetic improvement in sorghum. The germplasm being targeted by this study includes parental R-lines bred in Zimbabwe and collections of local sorghum lines. The panel is different from previously studied germplasm.

## 2. Results

### 2.1. Genetic Diversity Parameters

Genetic diversity parameters estimated using SNP-based KASP markers are presented in Table 1. The mean number of alleles per locus (*N_a_*) was close to two in all four groups (1.91 in the A-, R-lines and hybrids; 1.92 in the B-lines), consistent with the biallelic nature of the SNP markers. Average gene diversity (expected heterozygosity, *H_e_*) ranged from 0.23 in R-lines to 0.32 in hybrids (A-lines 0.28; B-lines 0.30). The higher He in hybrids reflects admixture of parental alleles, while the lowest He in R-lines indicates a genetic bottleneck due to selection for fertility restoration genes. The hybrids recorded the highest Shannon’s information index (*I*) of 0.48, followed by the B-lines (0.45), A-lines (0.42) and R-lines (0.37). The higher diversity in hybrids may reflect allele complementation from both parental groups, while the lowest diversity in R-lines suggests intensive selection for fertility restoration. Observed heterozygosity (*H_o_*) was uniformly low in the inbred lines (0.05, 0.01 and 0.03 for the A-, B- and R-lines, respectively) and substantially higher in the F_1_ hybrids (0.20); observed heterozygosity was therefore lower than expected heterozygosity in every group, consistent with the predominant homozygosity of the inbred parents. The inbreeding coefficient (*F_IS_*) was correspondingly high in the inbred lines (0.73, 0.96 and 0.87 for the A-, B- and R-lines, respectively) and lowest in the hybrids (0.41). The inbreeding coefficient FIS was highest in B-lines (0.96) and R-lines (0.87), which may reflect intense selfing during line development. A-lines showed moderately high FIS (0.73), which may be due to CMS conversion. In contrast, hybrids had the lowest FIS (0.41), which may indicate increased heterozygosity from inter-group crossing and confirming the genetic basis of heterosis. Average PIC values ranged from 0.19 in R-lines to 0.26 in hybrids, with intermediate values in A-lines (0.22) and B-lines (0.24). The higher PIC in hybrids may reflect greater allelic diversity and more balanced allele frequencies resulting from crossing the parents. The lowest PIC in R-lines may indicate fixation of alleles due to directional selection for fertility restoration, reducing marker informativeness.

### 2.2. Population Structure

**Figure 1:**
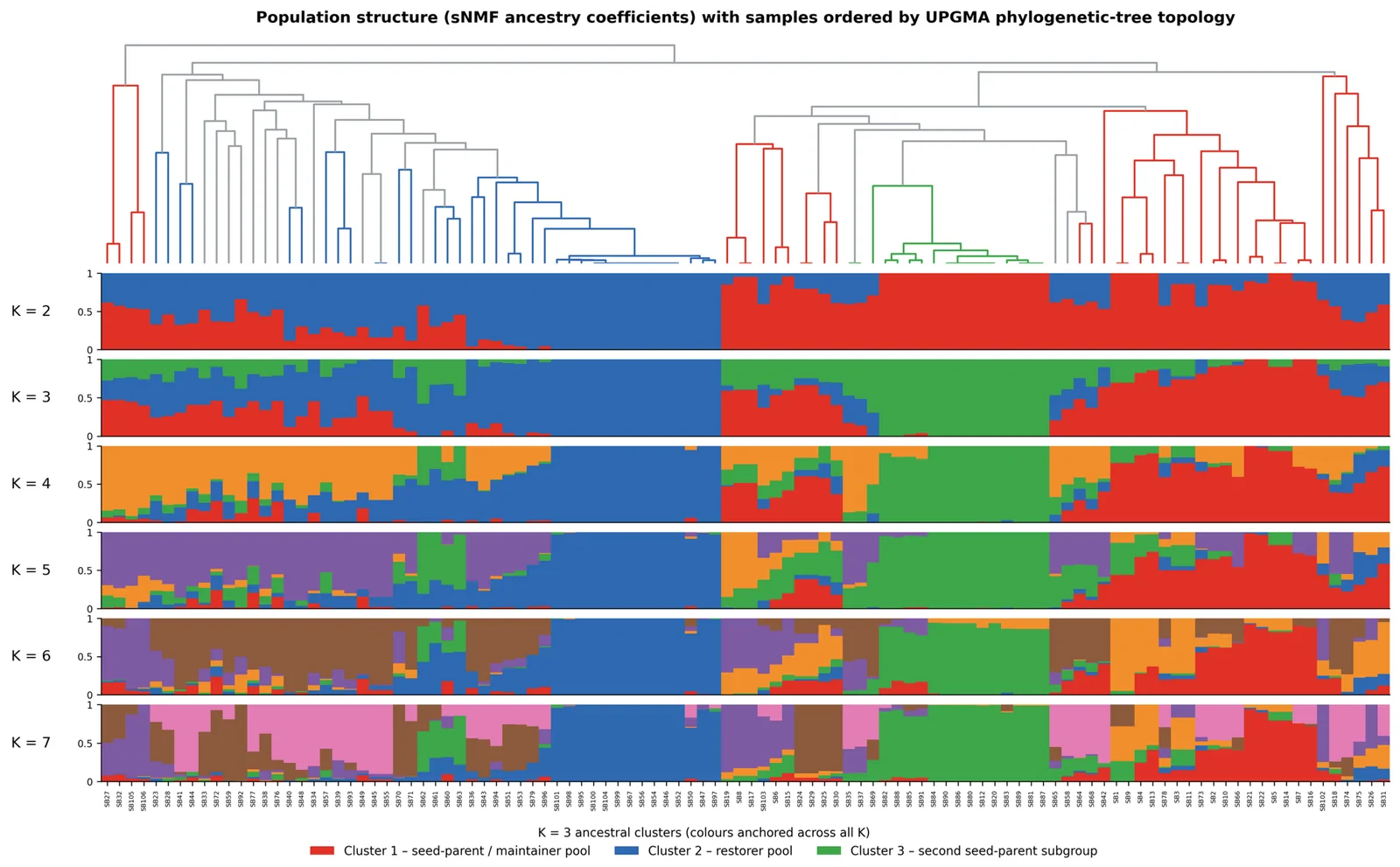
Ancestry coefficients for the 106 sorghum accessions across K = 2 to 7, estimated by sparse non-negative matrix factorization of the 79-marker allele-dosage matrix (an sNMF-type admixture analysis; Section 3.2). Each vertical bar is one genotype. K = 1 (the single-cluster null model) is included in the model-selection profiles (Figure S1). The UPGMA phylogenetic tree (simple-matching dissimilarity; the same topology as Figure 2) is displayed above the bar plots, and the genotypes are ordered along the x-axis according to the tree topology rather than by line type, so that the admixture profiles align directly with phylogenetic clustering. Colored segments give the estimated membership coefficient in each ancestral cluster (y-axis). To aid interpretation, the colors are anchored to the K = 3 solution and held consistent across all values of K, and the tree branches are colored by K = 3 cluster membership: red (Cluster 1, the seed-parent/maintainer pool), blue (Cluster 2, the restorer pool) and green (Cluster 3, the second seed-parent subgroup). The seed-parent (A-/B-line) pool separates from the restorer (R-line) pool at K = 2; a third component resolves at K = 3; and higher values (K = 4–7) subdivide these pools without changing the principal seed-parent/restorer structure, supporting K = 3 as the most parsimonious and biologically interpretable solution (cross-validated reconstruction-error and Gaussian-mixture BIC model-selection profiles are provided as Supplementary Figure S1). The four most divergent accessions (SB27, SB32, SB105 and SB106), which form dendrogram Cluster A, fall at the left-hand edge of the tree and appear as admixed individuals in the bar plots.

**Figure 2:**
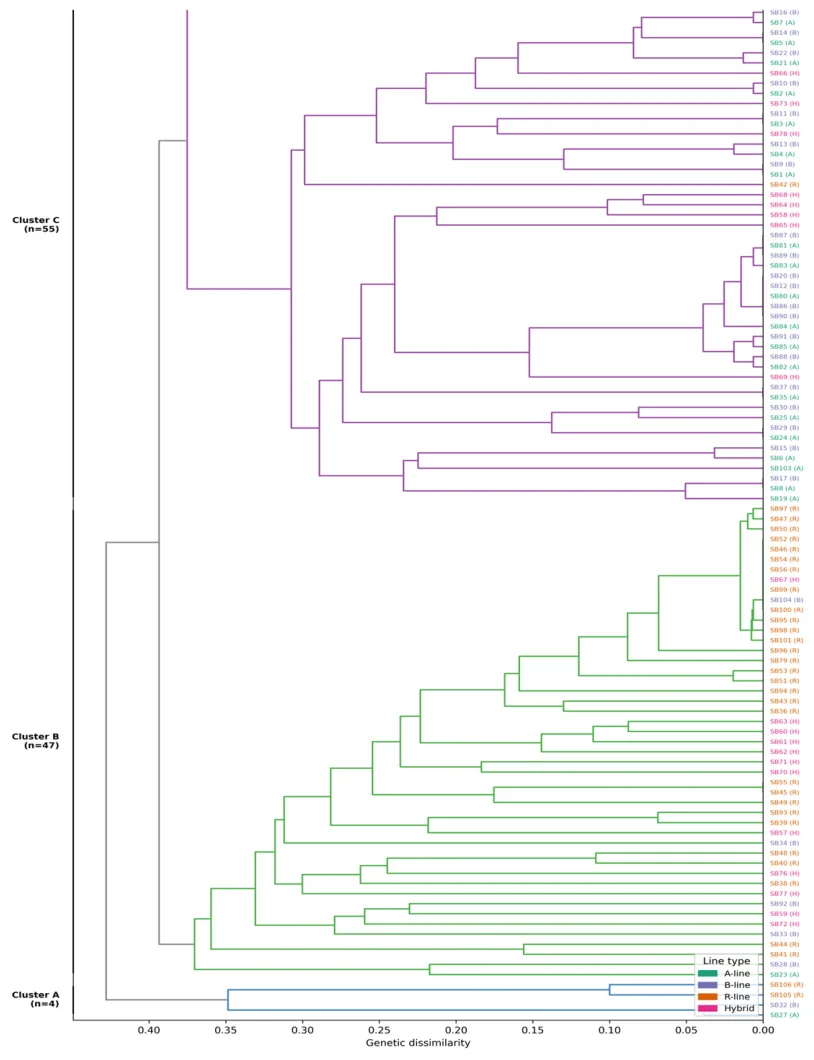
The dendrogram of 106 genotypes, according to hierarchical clusters based on 79 SNP-based KASP markers. Accessions were clustered by UPGMA from a simple-matching dissimilarity matrix and resolved into three clusters: Cluster A *(n* = 4), Cluster B (*n* = 47, restorer-dominated) and Cluster C (*n* = 55, seed-parent-dominated). Tip labels are colored by line type (A-, B-, R-lines and hybrids). The black lines shows the different clusters.

The population structure of the 106 evaluated sorghum accessions is presented in Figure 1. Across K = 1 to 7, the steepest reduction in cross-validated reconstruction error occurred up to K = 3, after which further increases in K produced only marginal improvement; this elbow, corroborated by the Gaussian-mixture BIC profile, supported K = 3 as the most parsimonious and biologically interpretable number of ancestral clusters (Supplementary Figure S1 and Table S5), consistent with the seed-parent versus restorer separation already evident at K = 2. At K = 3, the accessions resolved into three clusters, represented in red (Cluster 1), blue (Cluster 2) and green (Cluster 3). Cluster 1 (red) comprised 43 accessions (15 A-lines, 15 B-lines, nine hybrids and four R-lines) and represented a predominantly seed-parent (maintainer) pool. Cluster 2 (blue) comprised 41 accessions and was strongly dominated by restorer germplasm (28 R-lines, with nine hybrids, three B-lines and one A-line), representing a genetically divergent restorer pool. Cluster 3 (green) was the smallest group, with 22 accessions (10 B-lines, seven A-lines and five hybrids, and no R-lines), forming a second, distinct seed-parent subgroup. Thirty-seven accessions had a maximum membership coefficient below 0.6 and were therefore considered admixed; these included the four most divergent accessions, SB27, SB32, SB105 and SB106. Notably, A-line SB24 and its maintainer B-line SB29 were both assigned to Cluster 1, consistent with the near-identical nuclear genomes expected of a paired A-/B-line. These clusters correspond closely to the dendrogram clusters (Figure 2): the restorer-dominated Cluster 2 maps essentially in its entirety onto dendrogram Cluster B (restorer-dominated), whereas the seed-parent Clusters 1 and 3 together correspond to the maintainer-dominated dendrogram Cluster C; the four divergent accessions that form dendrogram Cluster A (SB27, SB32, SB105 and SB106) appear as admixed individuals within Cluster 1.

A genetic distance dissimilarity matrix showing pairwise divergence among the evaluated sorghum accessions is presented in Supplementary Table S4. The highest dissimilarity value of 0.59 was recorded between accessions SB92 and SB102, with comparably high values (≥0.55) for SB59 with SB102 (0.59), SB48 with SB106 (0.59), SB74 with SB106 (0.57), SB31 with SB41 (0.57), SB74 with SB105 (0.55), SB41 with SB102 (0.55) and SB19 with SB44 (0.55). Accession SB106 was the most divergent overall, showing dissimilarity values above 0.50 with 18 other accessions (including SB48, SB74, SB18, SB40 and SB44), while SB57 and SB102 each exceeded 0.50 with 14 and 12 accessions, respectively. The lowest dissimilarity value of 0.00, indicating accessions that were identical across the 79 SNP markers, was recorded for a group of eight mutually indistinguishable genotypes (SB46, SB52, SB54, SB56, SB67, SB99, SB100 and SB104), all of which showed pairwise dissimilarities of 0.00. Additional zero values were observed for accession SB90 with SB80 and SB86. The dendrogram grouped the studied sorghum accessions into three clusters (A, B and C) based on 79 SNP markers (Figure 2). Cluster A comprised four divergent accessions (SB27, an A-line; SB32, a B-line; and SB105 and SB106, both R-lines), accounting for 3.8% of the genotypes studied. Cluster B consisted of 47 genotypes (44.3%) and was dominated by restorer germplasm, comprising 29 R-lines (SB36, SB38–SB41, SB43–SB56, SB79 and SB93–SB101), 12 hybrids (SB57, SB59, SB60, SB61, SB62, SB63, SB67, SB70, SB71, SB72, SB76 and SB77), five B-lines (SB28, SB33, SB34, SB92 and SB104) and one A-line (SB23). Cluster C contained the majority of the genotypes (55 accessions; 51.9%) and comprised 22 B-lines, 21 A-lines, 11 hybrids and a single R-line (SB42).

The pairwise comparison of the evaluated sorghum accessions to show differentiation among the population clusters is presented in Table 2. Cluster A showed moderate and significant differentiation from Cluster B (*F*_st_ = 0.43; *p* < 0.01). High and significant differentiation was recorded between Cluster A and Cluster C (*F*_st_ = 0.34; *p* < 0.01). High and significant differentiation was recorded between Cluster B with Cluster C (*F*_st_ = 0.31; *p* < 0.01).

### 2.3. Analysis of Molecular Variance (AMOVA)

Analysis of molecular variance showing sources of variation among the evaluated sorghum accessions, comprised of 23 A-lines, 28 B-lines, 32 R-lines and 23 hybrids, is presented in Table 3. There were significant (Φ_ST_ = 0.55; *p* < 0.001) differences among and within populations of the studied sorghum accessions. Most of the molecular variance was partitioned almost equally between differences among the three dendrogram clusters (44.0%) and variation among individuals within clusters (45.2%), whereas differences among the breeding-line types (A-, B-, R-lines and hybrids) nested within clusters accounted for the smallest proportion (10.8%).

## 3. Discussion

### 3.1. Genetic Diversity Parameters

The 79 SNP-based KASP markers revealed variation across the genotyped sorghum A-, B-, R-lines, and hybrid accessions. Average gene diversity ranged between A and B for the studied sorghum accessions, recorded for hybrids (0.32), A-(0.28), R-(0.23) and B-lines (0.30). The range of between 0.23 and 0.32 indicates the presence of moderate genetic diversity, which may be useful to support breeding and selection strategies. A higher range in gene diversity was reported elsewhere. For example, gene diversity ranged between 0.1 and 0.5 in selected sorghum accessions genotypes using SNP markers [11]. Also, higher average gene diversity values of 0.53 and 0.57 were reported elsewhere in sorghum [20,26]. The relatively lower gene diversity observed in hybrids (i.e., 0.20) reflects the deliberate selection of genetically complementary but narrowly defined parental combinations in hybrid development programs, leading to reduced overall variation in the resulting progeny gene pool. Allelic richness in structured breeding populations is shaped by founder effects, genetic drift, and the intensity of selection applied during line development [27,28]. In the present study, the mean number of alleles per locus was close to two in all four breeding groups (1.91–1.92; Table 1), as expected for the biallelic SNP markers used. Comparable near-complete retention of both SNP alleles across breeding pools has been reported elsewhere [29]. The higher allelic richness was observed in B-lines (i.e., 1.92), which was similar to the findings of [30] when comparing B-lines to R-lines and landraces. The Shannon’s diversity index (*SI*) measures genetic diversity by considering both alleles and their relative frequencies across different levels of genetic organization, including loci, genotypes and populations. Higher *SI* values indicate balanced allele distribution and greater diversity at multiple genetic levels. On the other hand, values suggest dominance of a few alleles, reduced variation, and potential vulnerability within the population [24,31]. In the present study, the highest average *SI* value of 0.48 was recorded for hybrids, followed by B-(0.45), A-(0.42) and R-lines (0.37) (Table 1). The higher *SI* in hybrids verifies that hybrids capture a broader allelic diversity from genetically divergent parental lines. The high diversity observed in hybrids could contribute to better adaptability and yield stability. The relatively lower *SI* value for R-lines suggests that these accessions underwent the most intensive selection and genetic narrowing during the development of lines possessing specific restorer functions. Similar research also reported that Shannon’s diversity indices were lowest among accessions subjected to intensive selection in sorghum [32,33].

The average observed (*Ho*) and expected (*He*) heterozygosity averaged 0.05, 0.01, 0.03 and 0.20, and 0.28, 0.30, 0.23 and 0.32, respectively, for the A-, B-, R-lines and hybrids (Table 1). Observed heterozygosity was thus uniformly low in the inbred lines and markedly higher in the F_1_ hybrids. The uniformly low observed heterozygosity across the A-, B- and R-lines is consistent with their development as near-homozygous inbred lines through repeated selfing and backcrossing, which may progressively eliminate residual heterozygosity. The markedly higher observed heterozygosity of the hybrids suggests their F_1_ constitution, in which alleles contributed by genetically distinct parental lines are combined in the heterozygous state [34,35]. The discrepancy between observed and expected heterozygosity across all line categories further suggests the presence of inbreeding and population substructure within each breeding group, a pattern commonly reported in structured crop germplasm collections [36,37].

The differentiation coefficient (*Fst*) measures genetic variation among populations relative to total variation, with high values showing strong differentiation and low values indicating gene flow. Together with inbreeding coefficient *(F_IS_*) values, these statistics reveal the combined effects of genetic drift, selection, and population subdivision in shaping allele frequencies, reducing heterozygosity, and reinforcing structured differentiation across breeding groups [36,37]. The higher *F_ST_* in R-lines suggests greater allele frequency differentiation among restorer accessions, likely reflecting the diversity of geographic and germplasm sources from which restorer lines are typically assembled. The lower *F_ST_* values in hybrids than in R-lines is reflective of heterosis to restoring heterozygosity through recombination of diverse parental alleles. As a result, the high *F_IS_* values in R-lines reflect structured subdivision within restorer accessions, where distinct subpopulations have been developed independently for different hybrid combinations to create localized inbreeding within subgroups [38]. Regarding polymorphic information content (PIC), the average values ranged from 0.19 to 0.26 in R-lines and hybrids, respectively (Table 1). PIC values above 0.25 are generally considered moderately informative, while values above 0.50 are regarded as highly informative [39], indicating moderate marker discriminatory power across all genotype categories tested in the present study. Therefore, the lower PIC values in R- (i.e., 0.19) and A-lines (i.e., 0.22) (Table 1) suggest the opportunity to improve the KASP marker panels by introducing additional markers targeting highly polymorphic regions within evaluated sorghum breeding groups.

### 3.2. Population Structure Analysis

The use of the [40] delta K method to determine the optimum K value is a widely accepted approach that minimizes the risk of over- or under-estimating the number of true genetic clusters, and it has been extensively applied in sorghum population structure studies [41,42]. In the present study, population structure analysis identified an optimum of K = 3, delineating three genetically distinct clusters (Figure 1). This corresponds to the three-line classification of sorghum genotypes evaluated in the study, comprised of A-, B-, and R-lines, and hybrids. Similar research findings demonstrated that genomic characterization of structured sorghum germplasm consistently classify accessions into biologically meaningful clusters reflecting their breeding history and functional classification [43,44].

Cluster 1 comprised 15 A-lines, 15 B-lines, nine hybrids and four R-lines (Figure 1). The co-clustering of A- and B-line pairs is expected given that B-lines are the nuclear donors for corresponding A-lines, sharing identical or near-identical nuclear genomes differing only in cytoplasm [14]. The presence of nine hybrids within this cluster suggests these combinations were derived predominantly from parental lines within this group. Hybrid performance is maximized when parental lines are drawn from genetically divergent genetic backgrounds [45]. Therefore, the co-clustering of hybrids with their parental lines serves as a molecular indicator of potentially limited heterotic potential in the breeding pool, potentially due to insufficient genetic distance between parental combinations [46]. Cluster 2 was predominantly composed of R-lines (28 of 41 accessions) (Figure 1), confirming the expected genetic divergence of restorer lines from the maintainer and male-sterile line pools. The predominance of R-lines in the same cluster is an indication of a distinct genetic group, which could be a reflection of their restorer role and/or origin. A-line SB24 co-clustered with its maintainer B-line SB29 in Cluster 1 (Figure 1), as expected for a paired A-/B-line with near-identical nuclear genomes. Events (i.e., mutation, contamination, or involuntary outcrossing) reduce the integrity of the three-line system, necessitating the importance of routine molecular verification of breeding line identity. A similar lack of harmony between paired A- and B-lines was reported elsewhere in hybrid breeding programs, where molecular marker analysis revealed involuntary genetic changes during seed multiplication cycles [47]. The third cluster was the smallest grouping, comprising 22 accessions (10 B-lines, seven A-lines and five hybrids, with no R-lines) (Figure 1). This cluster represents a second, distinct seed-parent subgroup, consistent with shared ancestry among lines developed within the same breeding program over successive generations. The A- and B-line accessions of Cluster 3 represent additional maintainer (seed-parent) germplasm within the breeding program. The admixed accessions such as SB57, SB58, SB59, and SB103, which displayed membership coefficients across all three clusters, are genetically intermediate materials, which is common with the complex pedigree structures involved in hybrid breeding [19,27]. Given the clusters, breeding strategies could focus on Cluster 1 (red) to leverage A/B-line pairs for targeted hybrid development and use the R-lines from Cluster 2 and Cluster 3 for genetic distance to maximize heterosis. From Cluster 2 (blue), utilize R-lines for improving specific traits or broadening the genetic base and use them to develop hybrids by crossing them to A-lines from Cluster 3. In Cluster 3 (green), exploit the diverse gene pool for wider hybrid breeding or population improvement. These groupings can guide parent selection for maximizing heterosis and for conservation of diverse germplasm [42]. The structure analysis suggested three clusters reinforcing the dendrogram interpretation with the R-lines consistently resolved as the most divergent accessions whilst the A-lines and B-lines were fusing at a negligible distance.

### 3.3. Genetic Distance and Cluster Analysis

Genetic distance estimates derived from molecular markers are reliable predictors of heterosis in structured hybrid breeding programs, supporting the strategic use of highly dissimilar accession pairs as candidate hybrid parents [48]. In the present study, pairwise genetic distance analysis revealed a broad range of dissimilarity values, reflecting the diverse genetic backgrounds within the germplasm collection. The highest dissimilarity values exceeding 0.60 were recorded between SB19 and accessions SB105 (0.63), SB23 (0.62), and SB106 (0.63), as well as between SB60 and SB102 (0.62). These highly dissimilar pairs represent valuable candidates for divergent parental combinations in hybrid development, as genetic distance between parents is a primary determinant of heterosis [23,49]. Based on the present study, highly dissimilar accession SB6 (A-line) and SB105 (R-line) can be crossed to create a hybrid. Conversely, dissimilarity values of 0.00 between accessions SB13 and SB90 with genotypes SB80, SB84, SB86, and SB90, as well as between SB47 and SB48 with SB54, SB56, SB67, SB95, SB98, and SB99, indicate genetically identical or near-identical accessions. Identical or very similar lines can be used for parental development of A- and B-lines, which should be closely related [5]. For example, the B-line SB13 can be used to develop new A-lines or maintain genetically similar A-line accessions such asSB80 and SB84.

The dendrogram resolved the 106 sorghum accessions into three clusters (Figure 2). Cluster A comprised only four divergent accessions (SB27, SB32, SB105 and SB106; 3.8% of the collection), indicating genetic backgrounds distinct from the remainder of the panel. Cluster B comprised 47 accessions (44.3%) and was strongly dominated by restorer germplasm (29 R-lines), together with 12 hybrids, five B-lines and one A-line. The concentration of R-lines within Cluster B reinforces the genetic distinctiveness of the restorer pool and is consistent with the population structure results. Cluster C was the largest grouping (55 accessions, 51.9%) and comprised 22 B-lines, 21 A-lines, 11 hybrids and a single R-line (SB42), reflecting the shared genetic background typical of the maintainer and male-sterile lines developed through sequential backcrossing and selection. Pairwise comparison shows *F*_ST_ statistic and significance values among the genetic clusters of studied sorghum accessions (Table 2). There were significant differences at (*p* ≤ 0.01) between Cluster A and Cluster B, Cluster A and Cluster C, and Cluster B and Cluster C (Table 2).

### 3.4. Analysis of Molecular Variance

The AMOVA results revealed significant genetic differentiation (*Φ_ST_* = 0.55, *p* < 0.001), (Table 3) confirming that the SNP-based KASP markers possess sufficient discriminatory power to detect meaningful genetic structure within the collection. The largest proportion of variance was attributable to differences among the three clusters (44.0%) and among individuals within clusters (45.2%), which contributed almost equally, the smallest proportion being attributable to differences among the breeding-line types within clusters (10.8%) (Table 3). The large among-cluster component (ΦCT = 0.44) confirms that the three dendrogram clusters are meaningfully differentiated, while the comparably large among-individual component reflects the substantial genetic heterogeneity retained within each cluster, particularly Cluster C, which contains a mixture of line types with different breeding histories (Figure 2). Overall genetic differentiation was high and highly significant (ΦST = 0.55; *p* < 0.001). These findings are consistent with [24], who similarly reported substantial genetic differentiation among defined groups alongside a large within-group component in structured sorghum germplasm, attributing this to complex pedigree relationships and gene flow. The substantial within-individual component (45.2%) indicates that, although the three clusters are clearly differentiated, considerable genetic variation is retained among individuals within each cluster, consistent with the diverse pedigree origins represented in a structured breeding program. The notably high *F_ST_* value of 0.55 reflects the structured nature of the germplasm collection. Collectively, these AMOVA results provide robust statistical confirmation of genetic differentiation among the evaluated breeding line categories and support the strategic assignment of accessions to putative heterotic groups for hybrid parent selection. This is consistent with the frameworks proposed by [13] who demonstrated that molecular variance partitioning effectively guides heterotic group assignment in structured sorghum breeding programs. Also, [50] established that quantification of genetic differentiation among breeding pools through molecular marker-based variance analysis is a foundational step in the rational design of hybrid parent combinations in cereal crops.

## 4. Materials and Methods

### 4.1. Genetic Materials

One hundred and six (106) sorghum accessions sourced from the International Crops Research Institute for the Semi-Arid Tropics (ICRISAT) and Seed Co, Ltd, Harare, Zimbabwe gene banks were evaluated in the present study. These included 23 male-sterile A-lines, 28 B-lines, 32 R-lines and 23 hybrids (Table 4). All accessions were selected based on their adaptability to the rainfed growing conditions in Zimbabwe, which are characterized by mid-season droughts. The accessions were grown under rainfed conditions in an open field for characterization for grain yield and other agronomic traits, and for seed bulking. The 23 A-lines, 28 B-lines and four R-lines were selected from the ICRISAT drought nursery in Kiboko Kenya and introduced to Zimbabwe (Supplementary Table S1). The A-lines were selected based on male sterility, and the B-lines based on the ability to maintain A lines. R-lines were selected based on the ability to restore fertility in hybrids and adaptability to local conditions. The other 26 R-lines are Seed Co locally bred lines, two R-lines are local collections, and the 23 hybrids were constituted at Rattray Arnold Research Station in Zimbabwe. The hybrids were included in the study to assess their position in the population structure and understand how different they are from the parental lines genetically.

### 4.2. Genomic DNA Extraction

Seeds of the 106 sorghum genotypes were germinated in 2-L polyethylene pots under controlled greenhouse conditions at the Rattray Arnold Research Station (altitude 1341 masl, latitude 17°16′ S; longitude 31°03′ E). Leaves were harvested from each sorghum genotype 1 week after planting, for DNA extraction of leaf tissues using the modified cetyltrimethyl ammonium Bromide (CTAB) method [51].

### 4.3. Preparation of Reagents for DNA Extraction

Reagents for DNA extraction were prepared using the Seed Co standard operating protocol for DNA extraction of sorghum. The DNA extraction buffer was prepared in advance for optimal formulation and stability using 1 M tris-(hydroxymethyl) aminomethane HCl, 0.5 M, ethylenediamine tetraacetic acid (EDTA), double-distilled H_2_O, and sodium dodecyl sulfate (SDS). For the stock solutions, 157.6 g of tris-HCl (1 mol, molecular weight 157.6) was dissolved in 100 mL double-distilled water to make 100 mL of 1 M solution. EDTA (0.5 M, molecular weight 372 g, 18 g, 24 g and 612 g) was dissolved in 100 mL double-distilled water. The pH of tris-HCL and EDTA was first measured with a pH meter and then adjusted with HCl and NaOH pellets to a pH of 7.5 and 8.0, respectively. The 100 mL of 1 M tris-HCL, 100 mL of 0.5 M EDTA, 125 mL of 10% SDS, and 675 mL of distilled water were mixed to form 1 L of DNA extraction buffer. The mixture was properly prepared to ensure homogeneity, and stored in the incubator at 65 °C. A 6 M ammonium acetate solution was prepared by dissolving 924.96 g of ammonium acetate in 2 liters of double-distilled H_2_O water and refrigerated at 4 °C. To make 5 L of 70% ethanol, 3500 mL of absolute ethanol was mixed with 1500 mL of double distilled H_2_O.

### 4.4. DNA Extraction

Tungsten beads (1 × 6 mm) were added to each well of a 96-well plate using a bead dispenser after leaf sampling, and the plate was then sealed with silicon caps. The plates were placed into a Genogrider (Spex SamplePrepP, Metuchen, NJ, USA, 2010 series), which disrupts at 750 strokes for 3 min. Then, 500 μL of preheated DNA extraction buffer (tris-HCL, EDTA, and SDS) were added into each well, and plates sealed and shaken vigorously to mix the contents. The plates then were placed in an incubator at 65 °C for 45 min. After incubation, the plates were refrigerated to cool the samples to room temperature. Ammonium acetate was dispensed into the plates, which were then sealed and shaken vigorously for 15 s. The plates were refrigerated for 15 min and then centrifuged at 4000 rpm for 15 min. The supernatant liquid was aspirated and dispensed into storage plates containing a set amount of cold propane-2-ol using a fluid X liquid-dispensing machine. The propane-2-ol was prepared 15 min before this stage and stored in the refrigerator at 4 °C. The plates were then shaken and left to stand for 5 min, for DNA precipitation, after which the samples were centrifuged at 4000 rpm for 15 min to pellet the DNA. After centrifugation, the supernatant was tipped off using a quick flip motion without spilling liquid into other wells, and the plates were inverted on a paper towel to allow all the liquid to be drained off. The DNA pellets were then washed with 70% ethanol by dispensing 70% ethanol into the plates, centrifuging the plates, and discarding the ethanol to remain with the DNA. The plates were dried by incubation at 65 °C for 30 min. Lastly, 500 μL of water were added to re-suspend the DNA, then the plates were vortexed.

### 4.5. DNA Concentration Measurement

After extraction, DNA concentration was measured using NanoDrop. The NanoDrop was first calibrated to absolute zero before taking measurements. Four wells were randomly selected to extract 2 µL DNA from each. An average of the four wells was used as the sample’s DNA concentration. The DNA plates were then stored at −20 °C for further use.

### 4.6. DNA Dilutions and Drying

When preparing samples for polymerase chain reaction (PCR), the assay reaction requires 5–50 ng of high-quality DNA. Samples with high DNA concentrations were diluted at a ratio of 1 in 10 and those with low concentrations were diluted at a ratio of 1 in 5. For 1 in 10 dilutions, 180 µL of water was pipetted into dilution 96-well plates, with 20 µL DNA. For 1 in 5 dilutions, 160 µL of distilled water and 40 µL DNA was used. These were mixed to ensure an even distribution of DNA in the water. A fluid dispenser was used to transfer DNA from the dilution 96-well plates into the 384-well plates. Two 96-well sample plates were pipetted into a 384 PCR plate, with each plate representing a quadrant. Once the DNA had been dispensed, the plates were then centrifuged up to 2000 rpm, allowing DNA to settle at the bottom. The plates were then placed into an incubator at 65 °C and dried in the incubator for 1 h to evaporate all the water and remain with DNA only.

### 4.7. KASP Genotyping of Evaluated Sorghum Genotypes

Seventy-nine SNP-based Kompetitive Allele Specific (KASP) markers (Supplementary Tables S2 and S3) were used for the genotyping of evaluated sorghum genotypes, selected from Seed Co lines and introductions from ICRISAT. Genotyping was performed using 384-well PCR plates. The reaction mixture consisted of KASP Master Mix 514 µL plus water 514 µL to give a total of 1028 µL with Taq polymerase, MgCl_2_, dNTPs and ROX dye, 14.3 µL Assay Mix containing 2 allele-specific FAM/HEX labelled primers and glycerol as the buffer. The KASP markers used covered seven of the 10 sorghum chromosomes of sorghum (chromosomes 1–7); the panel did not include markers on chromosomes 8–10. Each KASP assay contained two allele-specific forward primers and one common reverse primer. The KASP Master Mix contained two Fluorescence Resonance Energy Transfer (FRET) cassettes, one labelled with FAM dye and the other with HEX/Vic dye. The dye enabled allele-specific fluorescence detection during genotyping. The genotyping mixture was dispensed into 384-well PCR plates with dried DNA samples using a Meridian machine. The plates were sealed with an optically clear seal using a Kube machine. The plates were centrifuged for up to 2000 rpm for 1 min.

The plates were placed in a Hydrocycler for 1-and-a-half hours or 35 cycles. The amplification stages were activation at 94 °C for 15 min, a touchdown phase or annealing 10 cycles at 55 °C, and regular cycling or denaturation at 95 °C to separate the DNA strands and extension at 72 °C for new strands. The chemical reactions that took place were primer binding of forward primers that perfectly matched the sorghum sample, DNA binding to SNP sites, amplification during PCR with the specific tail of the primer incorporated into the new DNA strand, and signal generation where the FRET cassettes in the Master Mix consist of fluorescent and quencher. When the tail sequence is amplified, the corresponding fluorescent dye (FAM or VIC) is released from its quencher and binds to the DNA, emitting a signal. After PCR, the plates are moved to a PHERAstar, which is a multi-mode micro-plate reader, to measure the intensity of FAM and VIC signals in every well. Results were visualized using a Polar or Cartesian cluster plot in KlusterCaller v3,2 software or application, with FAM (blue dye) on the x-axis, HEX/VIC (red dye) on the y-axis and a heterozygous mixture of both (green dye) in the middle.

### 4.8. Statistical Analysis

#### 4.8.1. Genetic Diversity Parameters

Genetic diversity parameters, including gene diversity, allelic richness (*R_s_*), Shannon’s diversity index, observed (*H_o_*) and expected (*H_e_*) heterozygosity, the differentiation (*F*_ST_) and inbreeding (*F*_IS_) coefficients, and polymorphic information content (PIC), were computed for each locus individually as well as for all loci combined (27), using GENEPOP (web version 4.0) [52] and FSTAT web version 2.9.4 (27). Gene diversity was computed according to the formula shown in Equation (1) below [53,54,55]:

(1) $$He=1-\sum_{i=1}^{k}Pi^{2}$$

where *H_e_* is the gene diversity (expected heterozygosity), *K* is the total number of alleles at that locus, AND *Pi* is the frequency of *i*th the allele.

The polymorphism information content (PIC) has the ability to detect the polymorphism among individuals in population [56]. PIC values were calculated as follows [57]:

(2) $$PIC=1-\sum_{u=1}^{k}\;{{\tilde{P}}^{2}}_{i}-\sum_{u=1}^{k-1}\;\sum_{j=+1}^{k}2{{\tilde{P}}^{2}}_{i}{{\tilde{P}}^{2}}_{j}$$

where; *k* is frequency of the marker allele, *k* is number of alleles, which is frequency of the *i*th marker, and is frequency of the *j*th marker. The PIC values were categorized as highly informative (PIC value of the marker >0.50), moderately informative (0.25 to 0.50) or slightly informative (<0.25) [58].

#### 4.8.2. Population Structure Analysis

Population structure was inferred from the 79 SNP-based KASP markers after encoding each genotype as an allele dosage (0, 1 or 2 copies of the minor allele) for the 106 accessions. Ancestry coefficients were estimated by sparse non-negative matrix factorization of the allele-dosage matrix (implemented in Python v3.14 with scikit-learn; non-negative matrix factorization with nndsvda initialization and a fixed random seed), an approach analogous to sparse non-negative-least-squares (sNMF) admixture estimation, for K = 1 to 7. The number of ancestral clusters was selected from the elbow in the cross-validated reconstruction error (five-fold cross-validation with 10% of matrix entries masked per fold), corroborated by the Bayesian Information Criterion of Gaussian-mixture models fitted to the leading principal components; both criteria indicated that resolving structure beyond K = 3 yielded only marginal improvement, so K = 3 was retained as the most parsimonious and biologically interpretable solution. Each accession was assigned to the cluster of maximal ancestry coefficient, and accessions with a maximum coefficient below 0.6 were regarded as admixed. The admixture bar plots and the model-selection profiles were produced with Matplotlib v3.9.2.

#### 4.8.3. Analysis of Molecular Variance (AMOVA) 

The components of total genetic variation, i.e., within and among populations, were tested using analysis of molecular variance (AMOVA) [59] in ARLEQUIN version 3.5.1.2 [60].

#### 4.8.4. Genetic Distance and Cluster Analysis

The genetic relationships between the 106 sorghum lines were described using the unweighted pair group method with arithmetic mean (UPGMA) cluster analysis using DARwin version 5.0.158 [61]. The minimal amount of valid data per marker was set at 70%, and 30,000 bootstraps were used to create the dendrogram. A dendrogram was generated based on the dissimilarity matrix, illustrating genetic distances between sorghum accessions. Pairwise comparison between genetic clusters as revealed by STRUCTURE analysis was also tested using ARLEQUIN version 3.5.1.2 at the 0.05 significance level.

## 5. Conclusions

This study aimed to test genetic diversity and population structure among 106 A-, B-, R-line accessions and hybrids using 79 SNP-based KASP markers. The KASP markers revealed moderate but informative genetic diversity across all genotype categories, with gene diversity ranging from 0.23 in R-lines to 0.32 in hybrids, providing breeders with a quantitative baseline for managing diversity within the three-line hybrid system. Population structure analysis at the optimum K = 3 delineated three functionally meaningful pools that may help in sorghum improvement. The 15 A-lines and 15 B-lines of Cluster 1 constitute a putative core maintainer pool, while the 28 R-lines of Cluster 2 represent a genetically divergent candidate restorer pool strategically suited for crossing with Cluster 1 A-lines to maximize heterosis. The smaller, R-line-free Cluster 3 forms a second seed-parent subgroup. Using SNP-based KASP markers, this study characterized diversity and genetic structure among the sorghum A-, B-, R-lines and hybrids used. Results suggests moderate, usable diversity and reveal clear separation into maintainer and restorer heterotic pools. The identified contrasting parental combinations can be prioritized to maximize heterosis, while genetically identical accessions can be rationalized to streamline the germplasm collection. These findings may provide a genetic framework to guide parent selection and hybrid breeding for climate-resilient sorghum improvement.

## Figures and Tables

**Table 1:** Genetic diversity parameters estimated for the study sorghum accessions genotyped using SNP-based KASP markers.

PIC	FIS	uHe	He	Ho	I	Ne	Na	N	Genotype Group
0.22	0.73	0.283	0.28	0.05	0.42	1.47	1.91	23	A-lines
0.24	0.96	0.305	0.30	0.01	0.45	1.50	1.92	28	B-lines
0.19	0.87	0.234	0.23	0.03	0.37	1.35	1.91	32	R-lines
0.26	0.41	0.328	0.32	0.20	0.48	1.56	1.91	23	Hybrids

N, number of accessions; Na, mean number of alleles per locus; Ne, effective number of alleles; I, Shannon information index; Ho, observed heterozygosity; He, expected heterozygosity (gene diversity); uHe, unbiased expected heterozygosity; FIS, inbreeding coefficient; PIC, polymorphic information content. Values are group means across 79 polymorphic SNP loci and all fall within the admissible range for biallelic SNP markers (0 ≤ He, Ho, PIC ≤ 0.5; I ≤ 0.69; −1 ≤ FIS ≤ 1).

**Table 2:** Pairwise comparison showing *F*_ST_ statistic and significance values among the genetic clusters of studied sorghum accessions.

Cluster C	Cluster B	Cluster A	
0.34 **	0.43 **	—	Cluster A
0.31 **	—		Cluster B
—			Cluster C

Significance levels; ** = *p* ≤ 0.01.

**Table 3:** Summary of AMOVA (analysis of molecular variance) comparing among and within 106 sorghum genotypes, which includes A-lines, B-lines, R-lines and hybrids according to 79 SNP markers.

Percentage Variation	Variance Components	Sum of Squares	Degrees of Freedom	Source of Variation
44.0	0.036	2.30	2	Among clusters
10.8	0.009	0.86	8	Among line-types within clusters
45.2	0.037	3.51	95	Individuals within clusters
	0.082	6.67	105	Total
0.55				Φ_ST_:
0.001				*p*-value:

**Table 4:** Information of sorghum accessions evaluated in the present study.

Entries	Genotypes Codes	Genotypes Descriptions
23	SB1, SB2, SB3, SB4, SB5, SB6, SB7, SB8, SB19, SB21, SB23, SB24, SB25, SB26, SB27, SB35, SB80, SB81, SB82, SB83, SB84, SB85, SB103.	A-lines
28	SB9, SB10, SB11, SB12, SB13, SB14, SB15, SB16, SB17, SB20, SB22, SB28, SB29, SB30, SB31, SB32, SB33, SB34, SB37, SB86, SB87, SB88, SB89, SB90, SB91, SB92, SB102, SB104.	B-lines
32	SB36, SB38, SB39, SB40, SB41, SB42, SB43, SB44, SB45, SB46, SB47, SB48, SB49, SB50, SB51, SB52, SB53, SB54, SB55, SB56, SB79, SB93, SB94, SB95, SB96, SB97, SB98, SB99, SB100, SB101, SB105, SB106.	R-lines
23	SB18, SB57, SB58, SB59, SB60, SB61, SB62, SB63, SB64, SB65, SB66, SB67, SB68, SB69, SB70, SB71, SB72, SB73, SB74, SB75, SB76, SB77, SB78.	Hybrids

## Data Availability

The original contributions presented in this study are included in the article/Supplementary Material. Further inquiries can be directed to the corresponding author(s). Supplementary Table S6 containing raw data is included.

## Supplementary Materials

The following supporting information can be downloaded at: https://www.mdpi.com/article/10.3390/plants15162488/s1. Table S1: Type of accession and sources of origin (ICRISAT Kiboko Kenya) and Seed Co Zimbabwe; Table S2: Per-marker diversity statistics for 79 SNP-based KASP markers; Table S3: Sorghum KASP (SNP) markers list; Table S4: pairwise genetic dissimilarity matrix; Table S5: Membership_K = 3; Table S6: Raw data. Figure S1: Selection of the number of ancestral clusters K. (a) Cross-validation cross-entropy, showing an elbow at K = 3; (b) Gaussian-mixture BIC computed on principal components.
