# Supplementary material for: SNP-Based KASP Markers Reveal Genetic Diversity and Population Structure Among African Sorghum Breeding Lines and Hybrids
Source: Plants (Basel). 2026 Aug 17;15(16):2488. doi: 10.3390/plants15162488 (PMC13516683; doi:10.3390/plants15162488)
Supplement: Supplementary file 1 [file plants-15-02488-s001.zip › Supplementary Figure S1_ Selection of K=3.pdf]

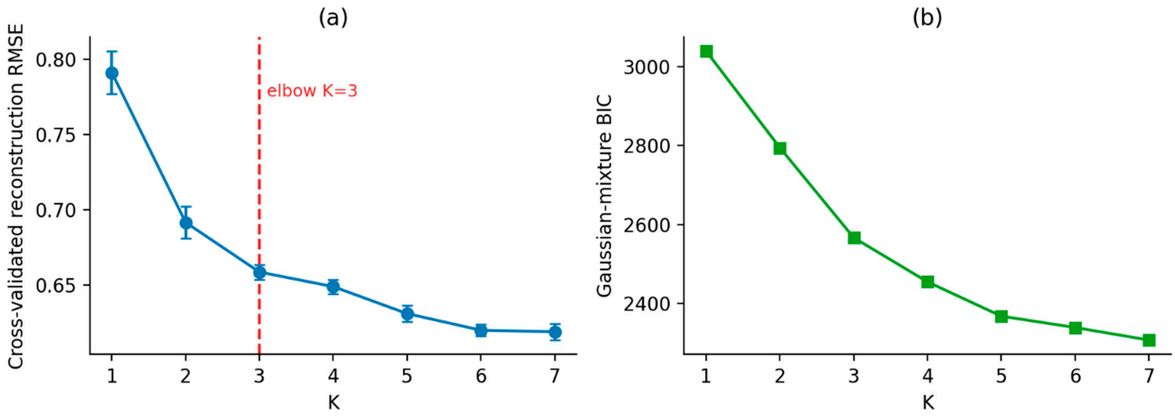

Supplementary Figure S1; Selection of the number of ancestral clusters K. (a) Cross-validation cross-entropy, showing an elbow at K = 3; (b) Gaussian-mixture BIC computed on principal components.

The cross-validation cross-entropy declined steeply from K = 1 to K = 3 and then plateaued, and the Gaussian-mixture BIC showed a comparable inflection, indicating that the major structure is captured by two to three ancestral clusters (Figure 3). The full admixture series from K = 1 to K = 7 is shown in Figure 5. At K = 2, the analysis cleanly separated the seed-parent pool from a restorer-dominated pool; at K = 3, a distinct hybrid-associated component emerged with hybrids carrying a mixture dominated by restorer ancestry combined with seed-parent background, the genomic composition expected of R × (A/B) crosses. Higher values of K progressively subdivided the pools without altering the principal seed-parent–restorer dichotomy.
