# Supplementary material for: SNP-Based KASP Markers Reveal Genetic Diversity and Population Structure Among African Sorghum Breeding Lines and Hybrids
Source: Plants (Basel). 2026 Aug 17;15(16):2488. doi: 10.3390/plants15162488 (PMC13516683; doi:10.3390/plants15162488)
Supplement: Supplementary file 1 [file plants-15-02488-s001.zip › Supplementary Table S1_ Sources of origin.pdf]

**Supplementary Table S1. Type of accession and sources of origin (ICRISAT Kiboko Kenya) and Seed Co Zimbabwe**

| Code Name | Line type | Source |
|---|---|---|
| SB1 | A | ICRISAT |
| SB2 | A | ICRISAT |
| SB3 | A | ICRISAT |
| SB4 | A | ICRISAT |
| SB5 | A | ICRISAT |
| SB6 | A | ICRISAT |
| SB7 | A | ICRISAT |
| SB8 | A | ICRISAT |
| SB9 | B | ICRISAT |
| SB10 | B | ICRISAT |
| SB11 | B | ICRISAT |
| SB12 | B | ICRISAT |
| SB13 | B | ICRISAT |
| SB14 | B | ICRISAT |
| SB15 | B | ICRISAT |
| SB16 | B | ICRISAT |
| SB17 | B | ICRISAT |
| SB18 | Hybrid | Seed Co |
| SB19 | A | ICRISAT |
| SB20 | B | ICRISAT |
| SB21 | A | ICRISAT |
| SB22 | B | ICRISAT |
| SB23 | A | ICRISAT |
| SB24 | A | ICRISAT |
| SB25 | A | ICRISAT |
| SB26 | A | ICRISAT |
| SB27 | A | ICRISAT |
| SB28 | B | ICRISAT |
| SB29 | B | ICRISAT |
| SB30 | B | ICRISAT |
| SB31 | B | ICRISAT |
| SB32 | B | ICRISAT |
| SB33 | B | ICRISAT |
| SB34 | B | ICRISAT |
| SB35 | A | ICRISAT |
| SB36 | R | Seed Co |
| SB37 | B | ICRISAT |
| SB38 | R | Seed Co |
| SB39 | R | Seed Co |

| | | |
|---|---|---|
| SB40 | R | Seed Co |
| SB41 | R icrisat | ICRISAT |
| SB42 | R icrisat | ICRISAT |
| SB43 | R icrisat | ICRISAT |
| SB44 | R icrisat | ICRISAT |
| SB45 | R | Seed Co |
| SB46 | R | Seed Co |
| SB47 | R | Seed Co |
| SB48 | R | Seed Co |
| SB49 | R | Seed Co |
| SB50 | R | Seed Co |
| SB51 | R | Seed Co |
| SB52 | R | Seed Co |
| SB53 | R | Seed Co |
| SB54 | R | Seed Co |
| SB55 | R | Seed Co |
| SB56 | R | Seed Co |
| SB57 | Hybrid | Seed Co |
| SB58 | Hybrid | Seed Co |
| SB59 | Hybrid | Seed Co |
| SB60 | Hybrid | Seed Co |
| SB61 | Hybrid | Seed Co |
| SB62 | Hybrid | Seed Co |
| SB63 | Hybrid | Seed Co |
| SB64 | Hybrid | Seed Co |
| SB65 | Hybrid | Seed Co |
| SB66 | Hybrid | Seed Co |
| SB67 | Hybrid | Seed Co |
| SB68 | Hybrid | Seed Co |
| SB69 | Hybrid | Seed Co |
| SB70 | Hybrid | Seed Co |
| SB71 | Hybrid | Seed Co |
| SB72 | Hybrid | Seed Co |
| SB73 | Hybrid | Seed Co |
| SB74 | Hybrid | Seed Co |
| SB75 | Hybrid | Seed Co |
| SB76 | Hybrid | Seed Co |
| SB77 | Hybrid | Seed Co |
| SB78 | Hybrid | Seed Co |
| SB79 | Hybrid | Seed Co |
| SB80 | A | ICRISAT |
| SB81 | A | ICRISAT |
| SB82 | A | ICRISAT |
| SB83 | A | ICRISAT |
| SB84 | A | ICRISAT |

| | | |
|---|---|---|
| SB85 | A | ICRISAT |
| SB86 | B | ICRISAT |
| SB87 | B | ICRISAT |
| SB88 | B | ICRISAT |
| SB89 | B | ICRISAT |
| SB90 | B | ICRISAT |
| SB91 | B | ICRISAT |
| SB92 | B | ICRISAT |
| SB93 | R | Seed Co |
| SB94 | R | Seed Co |
| SB95 | R | Seed Co |
| SB96 | R | Seed Co |
| SB97 | R | Seed Co |
| SB98 | R | Seed Co |
| SB99 | R | Seed Co |
| SB100 | R | Seed Co |
| SB101 | R | Seed Co |
| SB102 | B | ICRISAT |
| SB103 | A | ICRISAT |
| SB104 | B | ICRISAT |
| SB105 | R | Local Collection |
| SB106 | R | Local Collection |
