# Supplementary material for: SNP-Based KASP Markers Reveal Genetic Diversity and Population Structure Among African Sorghum Breeding Lines and Hybrids
Source: Plants (Basel). 2026 Aug 17;15(16):2488. doi: 10.3390/plants15162488 (PMC13516683; doi:10.3390/plants15162488)
Supplement: Supplementary file 1 [file plants-15-02488-s001.zip › Supplementary Table S2_ KASP Marker Information.pdf]

Supplementary Table S2. Per-marker diversity statistics for 79 SNP-based KASP markers

Computed from 106 sorghum accessions. MAF, minor allele frequency; CallRate, proportion genotyped; Ho, observed heterozygosity; He, expected heterozygosity; PIC, polymorphic information content.

Marker	Chromosome	Position	Alleles	MAF	CallRate	Missing	Ho	He	PIC
SB_01_001	1	1	2	0.108	1.000	0.000	0.066	0.193	0.175
SB_01_012	1	12	2	0.061	1.000	0.000	0.009	0.115	0.108
SB_01_024	1	24	2	0.428	0.981	0.019	0.106	0.490	0.370
SB_01_034	1	34	2	0.311	1.000	0.000	0.113	0.429	0.337
SB_01_049	1	49	2	0.467	0.991	0.009	0.114	0.498	0.374
SB_01_060	1	60	2	0.362	0.991	0.009	0.114	0.462	0.355
SB_01_072	1	72	2	0.162	0.991	0.009	0.000	0.271	0.235
SB_01_085	1	85	2	0.123	1.000	0.000	0.094	0.215	0.192
SB_01_096	1	96	2	0.216	0.915	0.085	0.000	0.339	0.282
SB_01_107	1	107	2	0.428	0.981	0.019	0.087	0.490	0.370
SB_01_120	1	120	2	0.467	1.000	0.000	0.123	0.498	0.374
SB_01_132	1	132	2	0.183	0.981	0.019	0.058	0.299	0.254
SB_01_156	1	156	2	0.419	0.991	0.009	0.076	0.487	0.368
SB_02_001	2	1	2	0.183	0.981	0.019	0.058	0.299	0.254
SB_02_018	2	18	2	0.479	0.887	0.113	0.000	0.499	0.375
SB_02_024	2	24	2	0.467	0.991	0.009	0.019	0.498	0.374
SB_02_030	2	30	2	0.457	0.981	0.019	0.125	0.496	0.373
SB_02_046	2	46	2	0.020	0.953	0.047	0.020	0.039	0.038
SB_02_062	2	62	2	0.075	1.000	0.000	0.019	0.140	0.130
SB_02_085	2	85	2	0.490	0.972	0.028	0.068	0.500	0.375
SB_02_092	2	92	2	0.284	0.981	0.019	0.125	0.406	0.324
SB_02_099	2	99	2	0.191	0.962	0.038	0.010	0.309	0.261
SB_02_104	2	104	2	0.381	0.953	0.047	0.129	0.472	0.360
SB_02_115	2	115	2	0.167	0.991	0.009	0.067	0.278	0.239
SB_02_135	2	135	2	0.269	0.981	0.019	0.019	0.393	0.316
SB_02_146	2	146	2	0.381	0.991	0.009	0.095	0.472	0.360

SB_02_164	2	164	2	0.439	1.000	0.000	0.047	0.492	0.371
SB_03_001	3	1	2	0.451	0.962	0.038	0.118	0.495	0.373
SB_03_001.1	3	1	2	0.111	0.981	0.019	0.048	0.197	0.177
SB_03_015	3	15	2	0.317	0.981	0.019	0.154	0.433	0.339
SB_03_029	3	29	2	0.386	0.991	0.009	0.143	0.474	0.362
SB_03_040	3	40	2	0.410	1.000	0.000	0.104	0.484	0.367
SB_03_052	3	52	2	0.151	1.000	0.000	0.038	0.256	0.223
SB_03_065	3	65	2	0.467	0.991	0.009	0.114	0.498	0.374
SB_03_082	3	82	2	0.010	0.991	0.009	0.000	0.019	0.019
SB_03_095	3	95	2	0.375	0.981	0.019	0.096	0.469	0.359
SB_03_101	3	101	2	0.019	0.981	0.019	0.000	0.038	0.037
SB_03_116b	3	116	2	0.429	1.000	0.000	0.104	0.490	0.370
SB_03_133	3	133	2	0.380	0.981	0.019	0.029	0.471	0.360
SB_03_169	3	169	2	0.338	0.962	0.038	0.049	0.448	0.347
SB_04_014	4	14	2	0.400	0.991	0.009	0.133	0.480	0.365
SB_04_026	4	26	2	0.024	0.972	0.028	0.010	0.047	0.046
SB_04_033	4	33	2	0.096	0.981	0.019	0.000	0.174	0.159
SB_04_039	4	39	2	0.014	0.981	0.019	0.010	0.028	0.028
SB_04_044	4	44	2	0.219	0.991	0.009	0.038	0.342	0.284
SB_04_081	4	81	2	0.243	0.991	0.009	0.029	0.368	0.300
SB_04_088	4	88	2	0.409	0.981	0.019	0.087	0.483	0.367
SB_04_096	4	96	2	0.333	0.991	0.009	0.057	0.444	0.346
SB_04_110	4	110	2	0.452	0.991	0.009	0.048	0.495	0.373
SB_04_120	4	120	2	0.349	1.000	0.000	0.057	0.454	0.351
SB_04_130	4	130	2	0.215	0.943	0.057	0.030	0.338	0.281
SB_04_141	4	141	2	0.100	0.991	0.009	0.029	0.180	0.164
SB_04_146	4	146	2	0.102	0.972	0.028	0.029	0.183	0.166
SB_05_002	5	2	2	0.250	0.981	0.019	0.058	0.375	0.305
SB_05_011	5	11	2	0.221	0.981	0.019	0.000	0.344	0.285
SB_05_019	5	19	2	0.345	0.915	0.085	0.072	0.452	0.350

SB_05_043	5	43	2	0.139	0.953	0.047	0.000	0.239	0.210
SB_05_068	5	68	2	0.216	0.962	0.038	0.078	0.338	0.281
SB_05_095	5	95	2	0.448	1.000	0.000	0.066	0.495	0.372
SB_05_108	5	108	2	0.443	0.991	0.009	0.124	0.493	0.372
SB_05_114	5	114	2	0.467	0.991	0.009	0.076	0.498	0.374
SB_05_129	5	129	2	0.294	0.962	0.038	0.098	0.415	0.329
SB_05_138	5	138	2	0.210	0.991	0.009	0.095	0.331	0.276
SB_05_155	5	155	2	0.148	0.991	0.009	0.048	0.252	0.220
SB_05_166s	5	166	2	0.278	1.000	0.000	0.160	0.402	0.321
SB_06_002	6	2	2	0.485	0.972	0.028	0.194	0.500	0.375
SB_06_011	6	11	2	0.033	1.000	0.000	0.009	0.064	0.062
SB_06_015	6	15	2	0.328	0.962	0.038	0.108	0.441	0.344
SB_06_031	6	31	2	0.033	1.000	0.000	0.047	0.064	0.062
SB_06_049	6	49	2	0.413	0.981	0.019	0.135	0.485	0.367
SB_06_060	6	60	2	0.295	0.991	0.009	0.095	0.416	0.330
SB_06_076	6	76	2	0.019	0.981	0.019	0.000	0.038	0.037
SB_06_090	6	90	2	0.086	0.991	0.009	0.057	0.157	0.144
SB_06_107	6	107	2	0.085	1.000	0.000	0.019	0.155	0.143
SB_06_120	6	120	2	0.159	0.981	0.019	0.029	0.267	0.231
SB_06_137	6	137	2	0.033	1.000	0.000	0.028	0.064	0.062
SB_06_145	6	145	2	0.066	1.000	0.000	0.019	0.123	0.116
SB_07_013	7	13	2	0.044	0.962	0.038	0.010	0.084	0.081
SB_07_022	7	22	2	0.460	0.943	0.057	0.100	0.497	0.373
**Mean (n=79)**				**0.263**	**0.981**	**0.019**	**0.064**	**0.340**	**0.270**
